# Assessment of Inter-Reader Reliability of Fazekas Scoring on Magnetic Resonance Imaging of the Brain in Adult Patients with Sickle Cell Disease

**DOI:** 10.3390/diagnostics15070857

**Published:** 2025-03-27

**Authors:** Aoife M. Haughey, Roisin M. O’Cearbhaill, Stephanie Forté, Joanna D. Schaafsma, Kevin H. M. Kuo, Igor Gomes Padilha

**Affiliations:** 1 Neuroradiology, Joint Department of Medical Imaging, University Hospital Network, University of Toronto, Toronto, ON M5T 2S8, Canada; roisinocearbhaill@gmail.com; 2 Division of Hematology and Oncology, Department of Medicine, Centre Hospitalier de l’Université de Montréal, Montreal, QC H2X 3E4, Canada; stephanie.forte.med@ssss.gouv.qc.ca; 3 Division of Neurology, Department of Medicine, University Health Network, University of Toronto, Toronto, ON M5T 2S8, Canada; joanna.schaafsma@uhn.ca; 4 Division of Medical Oncology and Hematology, Department of Medicine, University Health Network, Toronto, ON M5G 2C4, Canada; kevin.kuo@uhn.ca; 5 Division of Hematology, Department of Medicine, University of Toronto, Toronto, ON M5T 2S8, Canada; 6 Department of Radiology, School of Medicine, Queen’s University, Kingston, ON K7L 2V7, Canada

**Keywords:** sickle cell disease, magnetic resonance imaging, white matter disease, Fazekas scoring tool

## Abstract

**Background/Objectives**: Cerebral white matter disease is a common finding in patients with sickle cell that has been linked to cognitive impairment. However, there is no standardized approach for quantification of the cerebral disease burden. The Fazekas score is widely used to quantify the burden of white matter disease in chronic small vessel disease. However, its utility in sickle cell disease, specifically the inter-rater variability, has not been established. **Methods**: A patient cohort was compiled for the purpose of a research ethics board (REB)-approved retrospective study of adult patients with sickle cell disease, each of whom underwent MRI/MRA between the years 2017 and 2019. A total of 90 such patients were captured. All MRI/MRA studies were performed on three Tesla MRIs. Two independent neuroradiologists assessed the axial FLAIR MRI brain sequence (see image 1) for each of the 90 patients, with the sole focus of assigning a Fazekas score (0–3) to each study as a means of quantifying the burden of ischemic white matter lesions. The neuroradiologists were blinded to the scoring assigned by their counterpart and to the clinical information. After the initial assessment was completed, studies with discrepant Fazekas scores were documented and discussed by both readers. A consensus Fazekas score was then assigned to each of these studies. **Results**: Cohen’s weighted kappa was used as a measure of agreement between readers. The expected agreement was 74.65%, with an observed agreement of 94.44% between readers, with a kappa of 0.7808. **Conclusions**: We conclude on the basis of our study that there is good inter-reader reliability of Fazekas scoring on axial FLAIR MRI brain sequence in patients with sickle cell disease. The Fazekas is a promising measure that could easily be integrated in systematic evaluation of cerebrovascular lesions of adults with sickle cell disease.

## 1. Introduction

Sickle cell disease (SCD) is a monogenetic multisystem disorder with an autosomal recessive inheritance pattern. It is characterized by acute episodic illness and progressive multiorgan damage [1,2]. SCD is most prevalent in sub-Saharan Africa. It is estimated that approximately 515,000 children are born with the disease each year [3]. Recent figures suggest that there are more than 6000 patients living with SCD in Canada. Approximately 100,000 patients have SCD in North America [4].

Affected individuals inherit two copies of the sickle hemoglobin mutation (HbS), or they inherit one HbS mutation and one alternative globin gene mutation (HbC, β+-thalassemia and β0-thalassemia, for example) [5,6]. Prominent recognized factors that influence the severity of SCD include the genotype; for example, hemoglobin SC (Hb SC) is generally considered less severe [2]. Higher levels of fetal hemoglobin (Hb F) are considered protective of neurovascular injury [2,5]. The coinheritance of α-thalassemia may also be protective to some extent [2,7].

This multisystem disease manifests with sequelae driven by complex pathophysiological processes including vaso-occlusive episodes with associated ischemia and reperfusion injury, ongoing hemolytic anemia, a prothrombotic state, and chronic inflammation [8,9,10,11]. It has been long established that patients with SCD are at substantially increased risk of neurovascular complications. Up to 39% of pediatric patients with SCD suffer a silent cerebral infarct by the age of 18 [12]. Up to 11% of patients with SCD have clinically apparent strokes prior to 20 years of age. The risk increases to approximately 24% by age 45 [13].

Studies now increasingly suggest a robust link between neurocognitive disorders and white matter disease (WMD) related to anemia and hypoxia [14,15]. Studies have also established a correlation between the total volume of white matter disease and the severity of neurocognitive dysfunction, with lower full-scale intelligence quotient (IQ), Processing Speed Index (PSI), and Rowland Universal Dementia Assessment Scale (RUDAS) scores [16,17].

Given this growing evidence supporting the association between WMD or white matter connectivity and neurocognitive deficits in patients with SCD, it would be both desirable and practical to have a convenient and reliable means to quantify/grade WMD burden in this patient cohort. WMD quantification is a relevant and important parameter in diagnostic and treatment planning.

The American Society of Hematology now recommends at least one MRI as part of routine screening [18] in light of the high prevalence of cerebrovascular disease in patients with SCD. Standard reporting for quantifying imaging findings and long-term monitoring have not yet been defined.

The Fazekas tool has been long-established and accepted as a means for quantifying the burden of age-related WMD (ARWMD) on MRI [19].

It was developed to provide a simple, standardized, and reproducible method for assessing WMD, commonly seen in aging and neurological conditions such as small vessel disease and vascular dementia [19]. Given the strong association observed between WMD severity and cognitive decline, gait disturbances, and stroke risk, the Fazekas scale now plays a crucial role in stratifying disease burden, guiding clinical decisions, and supporting research on vascular cognitive impairment and dementia [20,21].

The utility of the Fazekas score in SCD, and specifically the inter-rater variability, has not been established. We conducted a retrospective study with the aims of (1) describing the Fazekas grade distribution in unselected adults with SCD and (2) measuring the inter-rater variability.

## 2. Materials and Methods

This is a retrospective cohort study. It has been approved by the research ethics board (REB), and participants signed informed consent.

### 2.1. Participants and Setting

Adults with SCD (any genotype), followed at the Comprehensive Red Blood Cell Disorders Clinic at the University Hospital Network in Toronto were eligible if (1) they had a magnetic resonance imaging study (MRI)/magnetic resonance angiography (MRA) examination performed between the years 2017 and 2019 as part of routine surveillance and (2) if they had signed informed consent. All such patients were included.

### 2.2. Patient Characteristics

Age, sex, and genotype were extracted from medical records.

### 2.3. Imaging

All imaging was reviewed to ensure patients had a 2D-FLAIR sequence performed as part of their examination. All patients had at least one 3T MRI performed at our institution, with most patients scanned according to a dedicated MRI/MRA protocol on either a Signa HDxt (GE Healthcare, Chicago, IL, USA) or a Skyra (Siemens Healthineers AG, Erlangen, Germany). Studies were acquired without intravenous contrast. Standard protocol included sagittal T1-weighted TSE sequence, axial T2-weighted TSE sequence, time-of-flight three-dimensional multi-slab MRA, axial fat-saturated T2-weighted fluid inversion recovery sequence (FLAIR), axial diffusion-weighted sequence b0 and b1000, and axial T2-weighted susceptibility weighted sequence.

### 2.4. Interpretation

Two independent neuroradiologists, with 2 and 4 years of clinical experience, respectively, assessed only the axial FLAIR MRI brain sequence for each of the ninety patients, with the sole focus of assigning a Fazekas score (0–3) to each study as a means of quantifying the burden of ischemic WMD [19] (see Table 1). The neuroradiologists were blinded to the scoring assigned by their counterpart and to the clinical information.

After the initial assessment was completed, studies with discrepant Fazekas scores were documented and discussed by both readers. A consensus Fazekas score was then assigned to each of these studies.

### 2.5. Statistics

Standard summary statistics were calculated using median [IQR] for continuous variables and proportions for categorical variables. Cohen’s weighted kappa was computed. Statistical analyses were performed on StataSE version 18. The expected agreement was 74.65%.

## 3. Results

Out of the 1100 patients with SCD followed at UHN, 90 patients had an MRI/MRA between 2017 and 2019 including FLAIR T2-weighted sequence and met the inclusion criteria. There were 46 male (51.1%) and 44 female (48.9%) patients. The median patient age was 28.0 years [IQR = 18].

**Figure 1:**
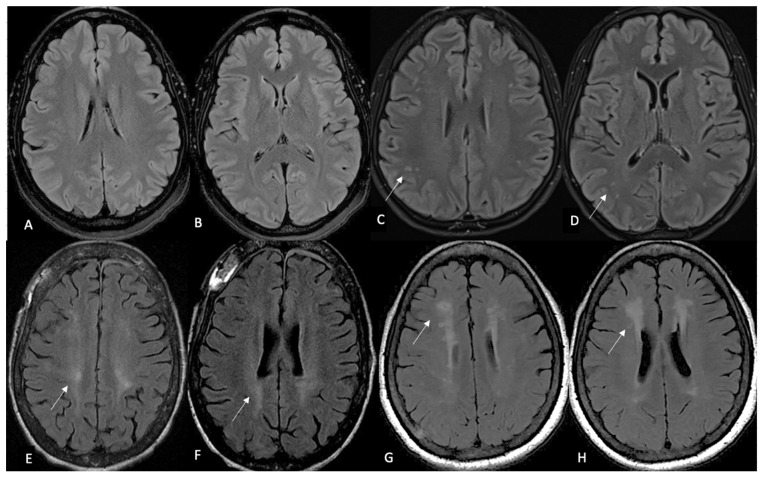
Multiple axial (Ax) T2-FLAIR MR images (**A**–**H**) depicting Fazekas scores 0–3. (**A**,**B**): demonstrate Fazekas 0, with no evidence of WMD; (**C**,**D**): show rare focal WM hyperintensities (white arrows), Fazekas 1; (**E**,**F**): show beginning of confluence of WM hyperintensities (white arrows), Fazekas 2; (**G**,**H**): show diffuse involvement of periventricular WM (white arrows), Fazekas 3.

The distribution of Fazekas scores (Figure 1) was 40.0% Fazekas 0 (36 of 90 patients), 51.1% Fazekas 1 (46 of 90 patients), 5.6% Fazekas 2 (5 of 90 patients), and 3.3%% Fazekas 3 (3 of 90 patients). The median Fazekas score was 1 with an interquartile range of 0–2.

**Figure 2:**
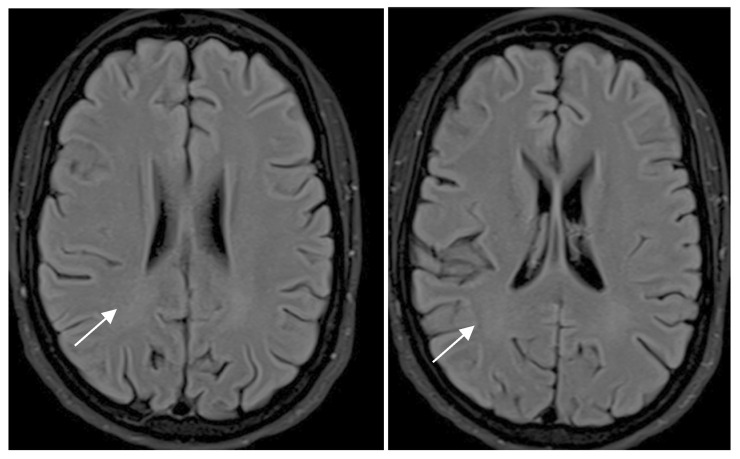
Ax T2-FLAIR MR images demonstrate hazy T2-FLAIR signal hyperintensity (arrows) in the posterior periventricular hyperintensity, presumed to be related to technical differences or technical artifacts. In total, 9 such cases were initially classified as Fazekas 0 and 2, respectively, by each of the readers, with consensus opinion of Fazekas 0.

Cohen’s weighted Kappa was used as a measure of agreement between readers. The observed agreement was 94.4% between readers, with a weighted Kappa of 0.79. Those that had discrepant initial reads/ disagreements consisted of 4 cases initially classified as Fazekas 0 and 1 by each of the readers respectively, with consensus read of Fazekas 1, 9 cases initially classified as Fazekas 0 and 2 respectively by each of the readers, with consensus opinion of Fazekas 0 (Figure 2), and 1 patient initially classified as Fazekas 1 and 2 respectively, with consensus read of Fazekas 1, with chronic lacunar infarct.

In the group of nine patients in whom there were initial discrepant reads of 0 and 2, respectively, it was noted that while the consensus read was that of Fazekas 0, the periventricular white matter demonstrated subtle hazy FLAIR signal hyperintensity, presumed to be related to technical differences or artifacts.

## 4. Discussion

Quantification of white matter lesions in adults with SCD using Fazekas confirmed that a high proportion of adults without overt neurological disease had some elements of vascular injury, mostly grade 1. To our knowledge, no studies have yet explored the utility of the Fazekas score in the context of SCD.

Our findings indicate excellent inter-rater reliability when applying the Fazekas score to this patient cohort. Until now, a visual rating scale for white matter disease (WMD) in SCD has been notably absent.

It is well established that patients with sickle cell disease (SCD) face a significantly increased risk of neurological complications, including cerebrovascular disease, vasculopathy [22], moyamoya-like syndrome [23], both overt and silent infarctions, with the latter being reported in up to 39% of pediatric patients by 18 years of age and in over 50% of patients by age 30 [24,25], brain hemorrhage, white matter disease and microstructural injury, global developmental delays, and neurocognitive difficulties [26,27].

WMD evident as white matter T2/FLAIR hyperintensities (WMH) on MRI are distinct from silent or overt cerebral infarcts. According to the American Society of Hematology, a silent cerebral infarct (SCI) is defined as a T2/FLAIR hyperintense lesion ≥ 3 mm in two planes identifiable on MRI that shows no corresponding findings on neurological examination [28]. White matter hyperintensities and SCI are often considered silent ischemic injury because no overt neurological sign is apparent. Conversely, overt cerebral infarction refers to an acute neurological injury resulting from ischemia or hemorrhage that persists for more than 24 hours [28], with both imaging evidence of an infarct and associated clinical symptoms.

Van der Land et al. [16,29] demonstrated that a greater burden of WMD on T2/FLAIR MRI sequences correlates with lower full-scale IQ scores, poorer processing speed indices, and increased fatigue in patients with SCD. They suggest that development of WMD is linked to endothelial dysfunction and reduced cerebral blood flow (CBF).

Schatz et al. [30] examined the relationship between IQ and MRI findings in a cohort of children with SCD. They found that patients with a large burden of SCI achieved significantly lower IQ scores, as measured by the Wechsler Full-Scale IQ test, compared to those with smaller burdens or none at all. Recent studies [14,16,17,31] have found that widespread white matter abnormalities were significantly associated with slower processing speeds, even in the absence of established SCI or overt infarcts on MRI.

Given the extensive research underway regarding the causes and clinical effects of WMD in patients with SCD—and the growing evidence linking WMD to cognitive impairments in this population—we believe the Fazekas tool has merit as a potential means of quantifying WMD burden. The Fazekas tool is long-established and accepted as a means for quantifying the burden of age-related WMD (ARWMD) on MRI.

Before the Fazekas scoring tool was developed, it was noted that the severity of age-related white matter disease correlated with cognitive decline, gait disturbances, and stroke risk [19,20,21]. This provided the rationale for creating a systematic grading system for WMD to assess extent and for potential clinical implications. The need for a simple, reproducible, and visual scale was identified. Prior to its advent, assessment for WMD burden lacked a standardized grading system. A visual scoring system (ranging only from 0 to 3) makes the Fazekas scoring system easy to apply in the clinical and research setting. The scoring system was designed to be reproducible across observers and imaging protocols. The latter is very important, allowing for more uniform reporting of radiological studies more broadly.

This is also pertinent and applicable to the SCD population. Acknowledging the correlation between the burden of WMD in SCD patients and the risk of cognitive decline [15,16,17,29], it is imperative that we have a reliable and reproducible tool to grade the WMD severity, which may in turn facilitate management decisions.

This scoring system has not previously been utilized to assess WMD in patients with SCD. This visual grading system, utilizing a 4-point scale, demonstrates strong inter-rater agreement.

A significant advantage of the Fazekas scoring tool is its quick and employable approach to assessing WMD burden, especially when compared to more labor-intensive quantitative methods, such as volumetric analysis, arterial spin labeling, diffusion tensor imaging, and perfusion MRI. At present, conventional MRI (FLAIR, T2-weighted imaging) remains the standard for assessing white matter disease in SCD. The Fazekas scoring system is a simple and reliable tool, applied to standard axial T2-FLAIR sequences, whilst alternative techniques are not routinely available or practical for everyday use by radiologists. This scoring system is useful, practical, and readily employable.

It is essential for a scoring system to exhibit good intra- and inter-rater reliability in diverse clinical settings. We demonstrated an excellent inter-rater agreement.

### 4.1. Limitations

Our study succinctly addresses the research question in the target population. This is a retrospective review. Similar to previous studies assessing inter-rater reliability of ARWMD scores, we do not have histopathology to confirm our assumption that the WMD visualized on MRI truly represents WMD related to SCD. While this represents a limitation of our study, this does reflect normal daily practice.

We do not account for confounders in this study, such as coexisting hypertension, dyslipidemia, or leukoencephalopathies, such as CADASIL, CARASIL, etc. Cerebral small vessel disease is not specific to any one disease process. In the aging population, it is most often multifactorial, involving entities such as hypertension, dyslipidemia, and other comorbidities that can contribute to WMD. While we cannot entirely exclude these confounders in our study, it is also important to emphasize that our patient cohort is young. As such, hypertension and dyslipidemia or other more age-related confounders are less likely to be present in this cohort.

We have assessed imaging at one time point only. A prospective study assessing more than one time point per patient would be required to assess inter-rater reliability of the Fazekas score in determining the progression of WMD.

### 4.2. Future Directions

Prospective assessment of the correlation between the assigned Fazekas score on MRI and patient cognition would be useful. Standardized implementation of the Fazekas scoring tool in the interpretation of MRI brain images in patients with SCD would allow for more streamlined reporting and comparison with prior studies. Ultimately, future research involving multi-center studies and a prospective study design could further validate our findings and assess the applicability of Fazekas on a broader scale and the possible integration of the Fazekas scoring system into the routine evaluation of WMD in SCD.

## 5. Conclusions

Based on our study, we conclude that there is strong inter-reader reliability of Fazekas scoring on axial FLAIR MRI brain sequence in patients with sickle cell disease. This convenient scale could be applied systematically to standardize reporting of findings of microvascular cerebral disease in sickle cell disease. The utility of the Fazekas as a measure of disease severity and risk of further neurological damage requires further studies.

## Figures and Tables

**Table 1:** Table detailing the various grades of the Fazekas scoring tool.

White Matter Lesions	Rating Scale for MRI
0	No lesions
1	Focal lesions
2	Beginning confluence
3	Diffuse involvement of entire region, with or without involvement of U fibers

## Data Availability

The raw data supporting the conclusions of this article will be made available by the authors on request.

## Abbreviations

The following abbreviations are used in this manuscript:

SCD	Sickle cell disease
HbS	Sickle hemoglobin mutation
WMD	White matter disease
ARWMD	Age-related white matter disease
MRI	Magnetic resonance imaging
MRA	Magnetic resonance angiography
FLAIR	Fluid attenuation recovery
CBF	Cerebral blood flow
SCI	Silent cerebral infarction
CADASIL	Cerebral autosomal dominant arteriopathy with subcortical infarcts and leukoencephalopathy
CARASIL	Cerebral autosomal recessive arteriopathy with subcortical infarcts and leukoencephalopathy
